# A new focus of Brazilian spotted fever in the central-west region of São Paulo state, Brazil

**DOI:** 10.1590/0037-8682-0391-2020

**Published:** 2021-03-22

**Authors:** Felipe Fornazari, Cristianne Dantas Freirias, Heloísa Coppini de Lima, Mauricio Mariani Rodrigues, Helio Langoni, Carlos Roberto Teixeira

**Affiliations:** 1 Universidade Estadual Paulista Júlio de Mesquita Filho, Faculdade de Medicina Veterinária e Zootecnia, Departamento de Produção Animal e Medicina Veterinária Preventiva, Botucatu, SP, Brasil.; 2 Universidade Estadual Paulista Júlio de Mesquita Filho, Faculdade de Medicina Veterinária e Zootecnia, Centro de Medicina e Pesquisa em Animais Selvagens, Departamento de Produção Animal e Medicina Veterinária Preventiva, Botucatu, SP, Brasil.

**Dear Editor,**

Brazilian spotted fever (BSF) is one of the most important zoonotic diseases in Brazil, with lethality rates reaching 80% in patients with delayed treatment^1^. It is caused by the tick-borne bacterium *Rickettsia rickettsii*, transmitted mainly by the vectors *Amblyomma sculptum* and *A. aureolatum* ^2^. Capybaras (*Hydrochoerus hydrochaeris*) and opossums (*Didelphis aurita*) are known to be competent amplifier hosts of BSF^3^. High rates of capybaras seropositive for *R. rickettsii* have been found in areas with BSF cases^4^ ^,^ ^5^.

Notification of BSF is mandated by the Brazilian Health Ministry. According to the latest records available in the Notifiable Diseases Notification System^6^ database (SINAN, accessed in May 2020), Brazil reported 237 BSF cases in 2018. This number is high with respect to the Brazilian historical records, which have been below 170 cases per year^6^, thereby indicating that BSF cases have recently increased in Brazil.

**FIGURE 1:**
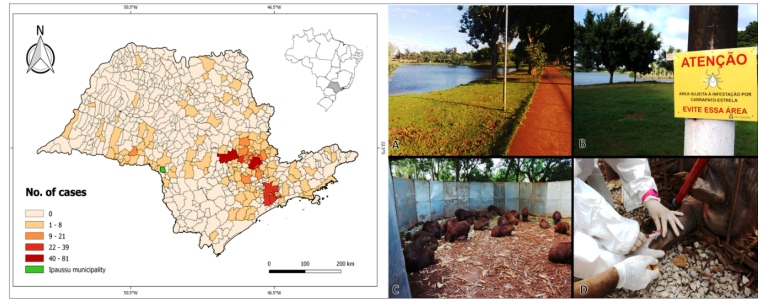
Left picture: Distribution of Brazilian spotted fever cases in São Paulo State municipalities, Brazil, between 2007 and 2017; Data were obtained from the Notifiable Diseases Notification System database (http://www.portalsinan.saude.gov.br, accessed in March 17, 2020. Cases from 2018 and 2019 stratified by municipality were not available. Data were retrieved considering the municipality the patient was living as the origin of transmission). The map was created in the QGIS 2.18 software using the graduated style with natural breaks (Jenks) algorithm. The green region indicates Ipaussu municipality. Right picture: Lake inside the urban area of Ipaussu municipality (São Paulo state, Brazil) inhabited by an estimated population of 60-70 free-ranging capybaras (*Hydrochoerus hydrochaeris*) **(A)**. Signposts warning about the risk of tick bites near the lake; **(B)**. Capybaras trapped inside a corral for chemical immobilization, identification with ear tags, and blood collection for serological tests **(C and D)**.

São Paulo state (SP), the most populated and developed state of Brazil, has the highest incidence of BSF cases^6^, with a majority of them concentrated in the central-east and south-east regions (Figure 1). According to SINAN, 104 (43.8%) of the 237 cases reported in 2018 in Brazil were from SP^6^, indicating a continuous demand for the surveillance and control of BSF in this state. As of January 2019, a series of BSF cases was notified in Ipaussu, a small town in the central-west region of SP that is not considered endemic for BSF and had no previous records in the SINAN database. The present report aims to highlight the emergence and geographical expansion of BSF in a new locality in SP as well as briefly describe the control actions implemented by the local government in collaboration with public institutions.

Ipaussu is a town, with approximately 15,000 inhabitants, located in a region with intensive agricultural land. Sugarcane crops prevail in the landscape and native forests are extremely scarce and fragmented, as they mostly fall in the SP territory. According to the Health Secretariat of Ipaussu (personal communication), 5 laboratory-confirmed cases of BSF were recorded between January 2019 and May 2020, with four of these cases resulting in the patients’ deaths. Since the beginning of the outbreak, the local health authorities have linked the emergence of this disease to a large lake located in the center of the city that is inhabited by an estimated population of 60-70 free-ranging capybaras, the most important host species of BSF (Figure 1). The animals were initially introduced in the 1980s, and their numbers have increased since then. Numerous people visit the lake daily as it is the main tourist point of the city, and due to its central location in the urban area, several Ipaussu citizens seem to cross this lake during their daily activities. In August 2019, the Superintendence of Endemics Control of SP collected ticks from the vegetation around the lake and identified them as *A. sculptum*, which is the main vector of BSF in the countryside of SP^2^ (*A. aureolatum* is the main vector in São Paulo city and its neighboring metropolitan areas). No other tick species were identified. In addition to capybaras, other primary hosts of *A. sculptum* such as tapirs (*Tapirus terrestris*) and horses are absent and scarce in Ipaussu, respectively. In October 2019 and January 2020, the epidemic gained immense attention from the media due to the death of three patients including boys aged 8-15 years^7^ ^,^ ^8^.

The local government implemented control measures by reducing the vegetation around the lake to decrease the tick infestation level, interdicting the lake to prevent people from accessing it, placing signposts near the lake about the risks of contracting BSF through tick bites, and orienting professionals from hospitals and healthcare units to stay alert about any suspicious case and to commence treatment immediately. Furthermore, blood samples were collected from the capybaras (Figure 1) to assess their level of exposure to *R. ricketsii* via serological tests and, thus, the need to control this animal population. All the procedures involving the capybaras were performed by the Center for Medicine and Wildlife Research of the School of Veterinary Medicine and Animal Science, São Paulo State University, and were authorized by the Secretariat for Infrastructure and Environment of SP (protocol DeFau/CMFS-IS N^o^ 23/2020). By May 2020, no further cases of BSF were registered in Ipaussu.

Controlling BSF is extremely complex and challenging, mainly due to the difficulties in eliminating the ticks from the environment and controlling the populations of the amplifier hosts. In the central-east region of SP, for instance, BSF has been endemic for several years despite intensive and continuous efforts to control it. In the following months, the population of capybaras in Ipaussu can be reduced or eradicated via surgical sterilization and/or euthanasia according to the criteria established by the SP Health Secretariat^9^.

The present article describes a new focus of BSF in SP, thereby reporting an increase in the geographical distribution of this disease. The five cases recorded in Ipaussu corroborate the low prevalence of BSF in three neighboring cities (Chavantes, Piraju, and Santa Cruz do Rio Pardo) (Figure 1), suggesting a similar eco-epidemiological scenario. Although laboratory diagnoses of capybaras are required to confirm their role in the epidemiology of BSF in Ipaussu, the broad evidence of this species as the main reservoir of *R. rickettsii* in Brazil strongly suggests that these animals contributed to the emergence of BSF in this town. Considering the complexity of BSF control, it is crucial for the local government of Ipaussu to develop continuous and long-term preventive actions.
